# Addressing Hazards from Unscheduled Novel Psychoactive Substances as Research Chemicals: The Case of U-50488

**DOI:** 10.7759/cureus.1914

**Published:** 2017-12-06

**Authors:** Zubair M Amin, Kerry Anne Rambaran, Steven W Fleming, Kevin Cho, Liza Chacko, Saeed K Alzghari

**Affiliations:** 1 Thomas J. Long School of Pharmacy & Health Sciences, University of the Pacific; 2 Department of Clinical Sciences, Keck Graduate Institute; 3 Reference Health Laboratories, Gulfstream Diagnostics; 4 Gulfstream Genomics, Gulfstream Diagnostics

**Keywords:** novel psychoactive substance, u-50488, synthetic opioid, opioids, toxicology, research chemical, nps

## Abstract

Increased experimentation with easily accessible synthetic opioids is posing a hazard to the public. We discuss one such synthetic opioid, U-50488. Much is unknown about U-50488; however, due to its structural similarity to U-47700, there is possible risk associated with its use. Curtailing the use of synthetic opioids such as U-50488 will require a concerted effort targeting multiple problems.

## Editorial

**Figure 1:**
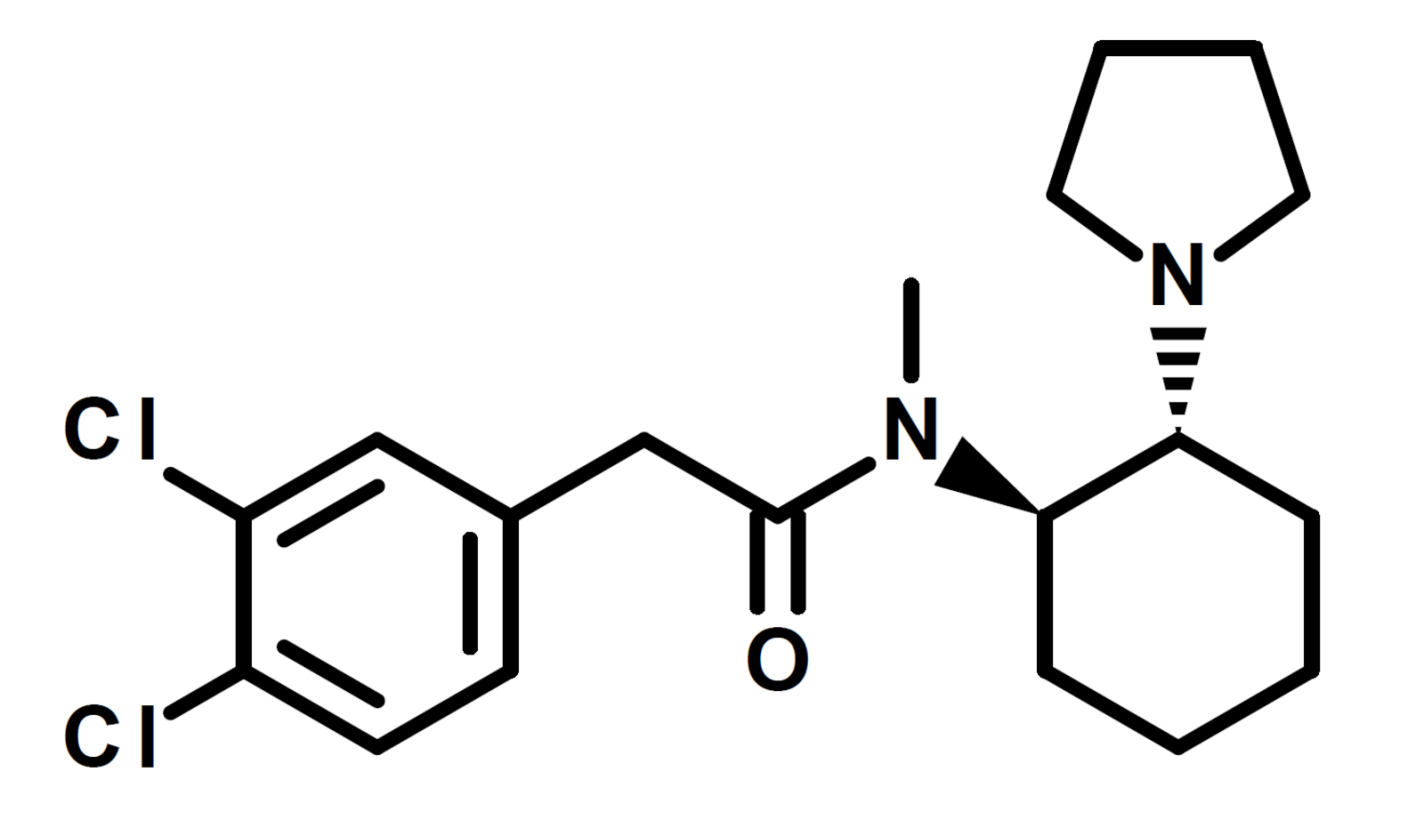
Structure of U-50488

The escalating opioid crisis continues to pose a threat to public health. In the United States, overdose deaths associated with opioids (including prescription opioids such as hydrocodone and illicit opioids such as heroin) have quadrupled since 1999. Furthermore, approximately 91 Americans die from opioid overdose each day [1]. The rise in the abuse of prescription opioids coupled with increased regulatory scrutiny has led to increased experimentation with unscheduled novel psychoactive substances (NPS) that are easily obtained online as “research chemicals.” We recently published articles highlighting the public health risks associated with two synthetic opioids that have been gaining prominence, U-47700 and U-49900 [2,3]. U-50488, possessing a similar molecular structure as U-47700, is another synthetic opioid that we believe requires increased scrutiny in the ever-growing catalog of NPS (Figure 1).

U-50488 is an opioid receptor agonist that was originally developed by the Upjohn Company in the 1970s as a κ-selective derivative of U-47700 [4]. Studies in animals have shown that U-50488 does not cause respiratory depression or constipation but it can cause diuresis and dysphoria [4]. While U-50488 was developed as a non-addicting analgesic agent, it never received FDA-approval nor has it been scheduled by the DEA.

To our knowledge, the only report evaluating the potential for U-50488 overdose in humans found no cases of its use in their analysis [5]. A growing phenomenon occurring on the Internet is the emergence of anecdotal accounts discussing the use of NPS labeled as “research chemicals” that individuals are purchasing through chemical manufacturers. Online forums including Reddit, Bluelight, and Flashback have pages dedicated to the use of NPS. While discussions of U-50488 are searchable on each of these forums, no anecdotal accounts of U-50488 use were found when these forums were queried.

Abuse of U-47700 led to 46 deaths before it was scheduled [2]. While much is unknown about the toxicological profile and toxicoepidemiology of U-50488, the structural similarity of U-50488 to U-47700 poses a risk. The ease of accessibility of U-50488 coupled with the potential risks associated with its abuse should serve as major causes of concern to clinicians and public health professionals. Clinicians should therefore be vigilant when it comes to this agent. Curtailing the use of U-50488 as well as other NPS may require a multi-pronged approach. This approach may consist of DEA scheduling to reduce the accessibility of this agent as well as increased focus on decreasing prescription opioid abuse as to deter the type experimentation that may lead one to abuse agents such as U-50488. Regarding the opioid crisis at-large, the DEA has implemented Operation Prevention to target grade-level students about the science behind addiction and create the dialogue to openly talk about the opioid epidemic. Continuing efforts to educate the public about this crisis need to span all walks of life that may not be aware of the threat of prescription and illicit opioids.
